# Comment on “ILC1 drive intestinal epithelial and matrix remodeling”

**DOI:** 10.1038/s41385-020-00360-9

**Published:** 2021-02-04

**Authors:** Fatima Hariss, Bertrand Meresse

**Affiliations:** Univ. Lille, Inserm, CHU Lille, U1286 - INFINITE - Institute for Translational Research in Inflammation, F-59000 Lille, France

## Abstract

Type 1 Innate lymphoid cells (ILC1) accumulate in the inflamed mucosa of patients with Crohn’s disease (CD) but their role in CD pathogenesis remains poorly known. In a recent issue of Nature materials, Jowett et al. (*Nat. Mat.* 2020) used a coculture model with intestinal organoids to show that ILC1 could promote intestinal epithelial growth and tissue remodeling through an unexpected mechanism that involves the transforming growth factor 1 (TGF-β1) and the metalloproteinase MMP9.

## Organoids, a « new » tool to dissect pathological immune responses in a dish

Over these past few years, organoid cultures have blown dust off in vitro studies. This is particularly true for the gut, where intestinal organoid culture has progressively replaced the traditional immortalized cell line-based systems (Caco-2, HT29-MTX…) and provides a more physiological model to investigate development and functions of the gut epithelium.^1^ The methodology initially developed by H. Clevers’ group,^2^ uses lgr5+ intestinal stem-cells (ISC) isolated from murine small intestinal crypts to recreate 3D crypt-villus structures (or organoid) that possess self-renewing capacity and generate differentiated epithelial cells (e.g., enterocytes, goblet cells, Paneth cells, enteroendocrine cells). Alternatively, induced pluripotent cells (iPS) generated from somatic cells can be differentiated into specific organ cell types in vitro. Intestinal organoids generated from human iPS create a more complex tissue containing both epithelial and mesenchymal lineages.^3^ Organoid formation requires cocktails of growth factors that support survival, self-renewal and differentiation of stem cells and an extracellular matrix (ECM) (often Matrigel), that provides a scaffold as well as signal cues important for cell attachment and development into 3D structures. These so called « mini-guts » are attractive platforms for modeling gut epithelium development, but also to study regeneration, metabolism and transport in intestinal epithelial cells^4^ as well as their interactions with infectious agents. Intestinal organoids are also useful for disease modeling as they can recapitulate in vitro genetic and epigenetic traits that influence pathological mechanisms. For example, it has been shown that the expression of several genes involved in antimicrobial defense (i.e., LYZ, PLA2G2A), epithelial secretory (i.e., ZG16, CLCA1) and absorptive functions (i.e., AQP8, MUC12) remains deregulated in colonic organoids from patients with ulcerative colitis (UC) but not from control individuals, indicating that organoids retain epigenetic imprinting.^5^ Finally, the organoid model provides a reductionist epithelial environment that can be enriched with other cell types in order to study their interactions in physiological and pathological contexts (e.g., infection, inflammation). This coculture model appears to be particularly helpful to dissect the pathogenic role of subsets of immune cells that infiltrate inflamed tissues. As an example, it has allowed to demonstrate that IFN-γ produced by T cells provokes epithelial damage by inducing Paneth cell death and loss of Lgr5+ ISC, a phenomenon observed in graft-versus-host disease, *Toxoplasma gondii* infection and autoimmune enteropathy.^6^

## Another arrow in the quiver of ILC1: intestinal ILC1 produces TGF-β1 and participates in tissue remodeling in CD

The recent article of Jowett and colleagues is one novel example of the interest of the organoid model to study pathological and protective mechanisms mediated by immune cells.^1^ In this study, the authors have investigated the role of ILC1 in CD and discovered their unexpected contribution to intestinal epithelial and matrix remodeling.

**Fig. 1:**
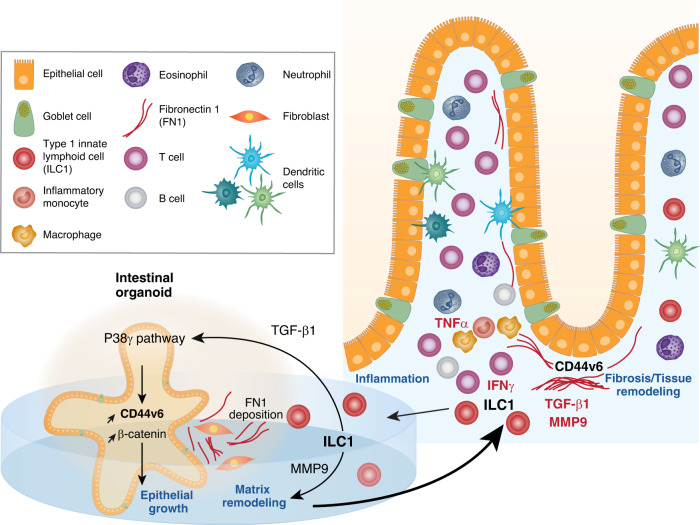
ILC1 influences intestinal epithelial growth and tissue remodeling through the secretion of TGF-β1. Crohn’s disease (CD) is a chronic inflammatory bowel disease of unknown cause. The pathological immune response is complex as it involves many cell types including inflammatory monocytes, neutrophils, dendritic cells, T and B cells as well as ILCs. The specific role in CD pathogenesis of each of them remains however unclear. Using a coculture model of intestinal organoids with ILC1 Jowett et al reveals an unexpected role of ILC1 in intestinal epithelial growth and tissue remodeling through a mechanism that involves TGF-β1 rather than IFN-γ. They show that TGF-β1 secreted by ILC1 stimulates the expression of the transmembrane glycoprotein CD44v6 on intestinal epithelial cells through the activation of the p38γ kinase and stabilizes β-catenin to promote the growth of intestinal organoids. The authors further show that ILC1 also increase CD44v6 expression on fibroblasts, degrade the MMP-sensitive matrix and drive fibronectin 1 deposition in fibroblast-rich, peri-organoid regions. Altogether these data show that ILC1 not only promote TH1-polarization in the inflamed mucosa but also participate to tissue remodeling and fibrosis in CD.

ILCs form a family of lymphocytes that, unlike T and B cells, lack surface expression of antigen-specific receptors. In the past decade, ILCs have attracted considerable interest and their development and functional properties have been extensively reviewed.^7^ Three distinct ILC populations (i.e., ILC1, ILC2 and ILC3) have been described that have distinct patterns of cytokine production and functions mirroring that of differentiated CD4 T helper (Th) cells.^7^ Type 1 ILCs have been defined by their capacity to produce IFN-γ. They include the conventional NK cells and T-bet dependent non-cytotoxic ILC1. In addition, owing to the plasticity of ILCs, ILC1 can also arise from either ex-ILC2 or ex-ILC3 which down regulate GATA3 or RORγt respectively and turn on expression of T-bet, and IFN-γ production. A protective role of ILCs has been demonstrated in many infectious models, notably at the early phase of infection. Conversely, ILCs may bolster or sustain pathological inflammation. Accordingly, ILC1 that arise from ILC3 expand in the inflamed intestinal mucosa during CD and are thought to participate in CD pathogenesis through their secretion of IFN-γ.^8^ The effects of ILC1 accumulation in the intestinal mucosa of CD patient remain however incompletely understood (Fig. 1).

Here, Jowett and colleagues took advantage of the organoid coculture model to decipher the impact of *lamina propria* ILC1 on the gut epithelium. By combining the coculture model with transcriptome analysis, they show an unexpected role of ILC1 in intestinal epithelial growth and tissue remodeling through a mechanism that involves TGF-β1 rather than IFN-γ (Fig. 1). Indeed, while ILC1 were expected to have deleterious effects on intestinal epithelial cells through their production of IFN-γ, Jowett et al show that murine intestinal organoids cultured for 4 days with murine intestinal ILC1 have enlarged CD44+ crypt buds indicating faster growing. They further show that ILC1 specifically stimulate expression of the CD44 variant 6 (CD44v6), a transmembrane glycoprotein mainly expressed in proliferative normal (e.g., intestinal crypts) and pathological (e.g., cancerous tumor, inflamed mucosa in IBD) tissues. CD44v6 is also known to influence cell proliferation and, as shown in a recent study, to stimulate epithelial migration and proliferation as well as wound healing in the murine model of DSS-induced colitis and in IBD.^9^ Moreover, Jowett et al observed in ILC1-organoid cocultures accumulation of epithelial β-catenin that colocalized with CD44v6 and evidence of activation of the Wnt/β-catenin pathway. Since Wnt/β-catenin signaling is crucial for stem cell renewal and because CD44 and Wnt/β-catenin pathways positively regulate each other in the gut, the authors hypothesize that ILC1 may thereby create a positive CD44v6/β-catenin feedback loop, which promotes epithelial cell growth. Jowett et al. further reveal the unforeseen capacity of activated ILC1 to secrete TGF-β1, an anti-inflammatory cytokine that also influences cell growth and differentiation. They also demonstrate that TGF-β1 secreted by ILC1 is responsible for CD44v6 upregulation and activation of the Wnt/β-catenin pathway in intestinal epithelial cells by adding a neutralizing antibody to the coculture. Based on the use of pirfenidone, a p38γ inhibitor, the authors conclude that TGF-β1 induces CD44v6 expression and stabilizes β-catenin through a noncanonical pathway involving the p38γ kinase. Of note however, this antifibrotic drug is a rather weak and unselective p38 inhibitor and further investigations will be useful to confirm this pathway. Importantly, the secretion of TGF-β1 by ILC1 and its impact on CD44v6 expression and epithelial growth were recapitulated in a humanized coculture model in which freshly isolated intestinal ILC1 from IBD patients were cultured with human intestinal organoids (Fig. 1). Here, Jowett et al. used iPS-derived gut organoids that they generated from a skin-fibroblast-derived human iPSC line (KUTE-4) in order to compare the effect of ILC1 isolated from inflamed and uninflamed tissues of patients with IBD with a standardized gut epithelium. Thereby, they could show that ILC1 from inflamed tissues proliferate and accumulate more in coculture and have a stronger effect on CD44v6 expression than ILC1 isolated from the uninflamed mucosa. Since iPS-derived gut organoids contain both epithelial and mesenchymal cells, the authors could show that ILC1 also increase CD44v6 expression on fibroblasts and, thus, that ILC1 also participate in mesenchymal remodeling. Since expression of ECM-remodeling genes and metalloproteinase (MMP)-dependent degradation of the matrigel were enhanced in coculture with ILC1, Jowett et al. further studied the impact of ILC1 on ECM. To do so, they replaced “classical” matrigel by a homemade hydrogel matrix, which allowed precise quantification of ILC1-mediated matrix degradation and stiffening. Using this new matrix, they could demonstrate that ILC1 do not only degrade the MMP-sensitive matrix but also drive fibronectin 1 deposition in fibroblast-rich, peri-organoid regions (Fig. 1).

## Conclusions

To date, the physiological and pathological functions of ILC1 have been exclusively associated with their capacity to produce high amount of IFN-γ. The study by Jowett and colleagues highlights a new function for intestinal ILC1 in tissue remodeling through TGF-β1 secretion. Further studies will be important to define whether this function of ILC1 is restricted to pathological inflammation and fibrosis in diseases such as IBD or is more generally involved in tissue repair alike IL-22 produced by ILC3 or IL-13 and AREG secreted by ILC2. Beyond this interesting finding, this study provides a novel elegant demonstration of the usefulness of the organoid coculture model to identify and study the specific role of immune cells in the pathogenesis of complex immune disorders.
